# Premature Conclusion Leads to Serious Legal Consequences

**DOI:** 10.1155/S106474499700032X

**Published:** 1997

**Authors:** Sebastian Faro


					Infectious Diseases in Obstetrics and Gynecology 5:209 (I 997)
(C) 1997 Wiley-Liss, Inc.
Premature Conclusion Leads to Serious
Legal Consequences
There appears to be confusion over the approach obstetricians should take in
managing the pregnant patient with BV. Screening all patients for BV and treat-
ment has not been shown to improve perinatal morbidity or mortality. Although
current data are encouraging, a cause and effect relationship has not been estab-
lished. Unfortunately, patients are led to believe that the presence of this condi-
tion (BV) can be easily corrected. A treatment modality has not been established
that can prevent abortion or preterm birth. Recently a patient was awarded $1.7
million in a legal case because she had a positive Gardnerella culture and her
physician did not treat her.
It is important that we, as obstetricians-gynecologists with a special interest in
infectious disease, inform the pregnant woman of what is actually known concern-
ing BV and pregnancy. The infectious diseases societies, IDSOG and IIDSOG,
should issue position papers and recommendations with regard to BV. The posi-
tion paper should make it very' clear that at the present time no available treatment
has been shown to prevent abortion or premature delivery. Such a position paper
may allow pregnant patients with BV to understand the nature of this condition,
what to expect from available treatment, and that recurrence is common. A posi-
tion paper may also aid lawyers in understanding that BV is a disruption of the
patient's vaginal ecosystem and not an infection. Therefore, treatment cannot cure
this condition but is directed at attempting to restore the vaginal ecosystem to a
healthy state.
Sebastian Faro, MD, PhD
Editor-in-Chief
Editorial

				

